# On the spot immunocapture in targeted biomarker analysis using paper-bound streptavidin as anchor for biotinylated antibodies

**DOI:** 10.1007/s00216-022-04161-w

**Published:** 2022-06-10

**Authors:** Christina Johannsen, Anam ul haq, Léon Reubsaet, Trine Grønhaug Halvorsen

**Affiliations:** 1 Section of Biochemistry and Molecular Biology, Department of Biosciences, University of Oslo, Oslo, Norway; 2 Section of Pharmaceutical Chemistry, Department of Pharmacy, University of Oslo, Oslo, Norway

**Keywords:** Antibodies, Tailor-made, Smart sampler, Pretreatment, Universal anchor, Biotin

## Abstract

The modification of an easily available resource like paper to circumvent expensive or intensive sample pretreatment could be the answer to sample analysis in resource-poor regions. Therefore, a novel on-paper device combining sample collection with affinity sample pretreatment is introduced here. Universal smart affinity samplers are produced by a simple KIO_4_-mediated oxidation of cellulose, which functionalizes the paper. This is followed by immobilization of streptavidin. Streptavidin serves as a universal anchor for biotinylated antibodies, enabling simple preparation of tailor-made affinity samplers. The functionality of the device was tested using a model protein (human chorionic gonadotropin, hCG) and biotinylated anti-hCG antibodies for affinity capture. In a laboratory setting, the performance was demonstrated, and a 14-fold increase of target binding compared to binding without bmAb was achieved. The recovery of hCG captured with bmAb-treated samplers was determined to be 33% and comparable to previously described affinity capture approaches. Application of the smart affinity samplers to human serum containing hCG showed an *R*^2^ of 0.98 (200–1000 pg mL^−1^), precision of ≤ 9.1% RSD, and estimated limit of detection of 65 pg mL^−1^. Although further optimization and validation are necessary prior to application to real samples in clinical settings, the potential of the device for use in determination of low abundant biomarkers in complex samples has been demonstrated.

**Supplementary Information:**

The online version contains supplementary material available at 10.1007/s00216-022-04161-w.

## Introduction

Globally, 45% of inhabitants live in rural areas. Of this rural population, a majority of 92% lives in less developed regions [1]. Resource-limited settings lack access to technical service and human workforce. To tackle this, goal 3 of the Sustainable Development Goals of the United Nations was introduced to ensure good health and well-being. Therefore, cost-effective technologies, which can enable local communities in developing nations to improve healthcare [2], food quality [3], and environmental safety [4], will play a key role in the future. While diagnosis is the first step to treat a condition, the drawback in monitoring and controlling diseases remains to be simple, affordable, and robust diagnostic tests [5]. Latest developments in diagnostics call for highly simplified and cost-effective yet sensitive ways for the detection of disease biomarkers. State-of-the-art reliable and sensitive diagnostics are based on immunoassays such as enzyme-linked immunosorbent assays (ELISA), Western blot, or mass spectrometric analyses [6]. However, access or availability to some of these sophisticated tools can be limited, especially in resource-poor regions [7]. Therefore, smart device formats which allow simple sample pretreatment, which can be used in places without extensive medical infrastructure and in rural areas, are of high interest [8]. One way to approach this is paper-based sensors and assays due to their low cost and simplicity of operation. Paper-based assays range from using nanoparticle materials and unique structures for detection via different techniques to novel biological species for recognition of biomarkers [9, 10]. One of the most used paper-based approaches is paper-based immunoassays, such as paper strips where antibodies coupled to gold nanoparticle indicate the presence of the target analyte due to an appearing test line [11]. Another approach is the use of dried blood spots (DBS). DBS have been around since the early 1960s and proven to be an advantageous technique due to convenient storage and transport, analyte stability, requirement of low sample amount, minimal invasive sample collection, relative safety, and the possibility of collection outside of a laboratory or special facility [12–15]. When using DBS, samples are transported by postal mail and processed for analysis upon arrival at a laboratory. One advantage of DBS is that it can be combined with a variety of analytical techniques depending on the analyte in question. Sometimes the process in the laboratory can be challenging and time-consuming, e.g*.*, when it comes to mass spectrometry-based protein analysis. That is why recently, sampling cards for blood and other matrices have been facilitated as smart samplers in several ways. These smart samplers allow the pretreatment to start already upon blood collection, thus saving valuable time and material in the preparation of the sample later on. Integration of sample pretreatment steps as, for example, tryptic digestion for bottom-up protein analysis [16–18] or affinity capture for sample clean-up [19, 20] on, for instance, cellulose [21] is feasible. Technically, biomolecules such as proteins can be immobilized on paper in three different ways: (1) through adsorption due to weak, non-covalent, intermolecular forces; (2) coupling via a bioaffinity tag; or (3) linked through covalent binding. Examples for immobilizing proteins on paper surfaces using adsorption are direct adsorption without further modification [22] or direct adsorption within a wax-printed ring, which enables immobilization of biomolecules in a defined area [23]. Immobilizing proteins by using bioaffinity tags is usually achieved by exploiting specific binding phenomena which exist in nature as amongst others avidin–biotin binding, protein A/G-antibody, genetically engineered protein affinity ligands, DNA hybridization, and aptamers [24]. Holstein et al. demonstrated, for example, the genetic fusion of affinity proteins to nitrocellulose [22]. Immobilization of proteins on paper using covalent binding can be achieved in several ways. The surface of the paper allows for the direct functionalization of the hydroxyl groups, thus allowing the covalent linkage to the protein. Modification of the said hydroxyl groups has been investigated as amongst others carboxylic acids [25, 26], epoxides [25], aldehydes [27, 28], or via silanization and subsequent 2-hydroxyethyl methacrylate-co-2-vinyl-4,4-dimethyl azlactone (HEMA-VDM) polymerization of the silanized paper surface [29]. While all of these approaches ensure immobilization of protein to paper, the subsequent analysis varies. In combination with mass spectrometric analysis, only a couple of examples are available to our knowledge: HEMA-VDM polymerization and subsequent immobilization of trypsin or monoclonal antibody (mAb) [16, 29, 30], HEMA polymerization and subsequent tosylation for covalent mAb immobilization [19] or covalent binding via oxidation of the cellulose hydroxyl groups and binding of trypsin [17, 18]. However, common for all the described mass spectrometric approaches is that a specific sampler needs to be produced for each specific target protein to be analyzed. Instead, by immobilizing streptavidin (SA) to the modified paper, versatility of the biotin-SA system can be utilized and a universal sampler for binding of different biotinylated molecules can be produced, applicable for subsequent analysis. Different ways of immobilization of SA specifically on paper are described as binding via genetic fusion of biotinylation tags [31], as SA agarose resin adsorbed to paper [32] or adsorbed to nitrocellulose by a simple spot and dry approach [33], but not yet in smart samplers for simplified sample preparation prior to LC–MS/MS analysis.

This paper therefore investigates the possibility of using streptavidin as universal anchor for simple immobilization of biotinylated proteins to cellulose paper for simplified sample preparation of proteins prior to LC–MS/MS analysis. More specifically here, we describe the use of KIO_4_-functionalized paper [21, 25] to bind SA: By oxidizing cellulose paper samplers, the formation of aldehyde groups allows covalent binding of SA via their amine groups. SA can in turn be used for non-covalent binding of biotinylated molecules such as antibodies, which in turn enable immunocapture. This contrasts with previously described paper-based immunoaffinity devices where the antibodies were covalently linked to HEMA-VDM-functionalized paper [19, 20]. In the study presented, filter paper is simply oxidized for a two-step immobilization of antibodies. To prove that the principle works, a model system for capture of the human chorionic gonadotropin (hCG) using biotinylated antibodies (bmAbs) targeting intact hCG was established. Captured hCG was subsequently subjected to reduction, alkylation, and tryptic digestion and analyzed by targeted MS/MS monitoring the intensity of the proteotypic peptide βT5. In this way, a completely new and generic strategy for possible remote sampling and targeted protein determination using smart affinity samplers is generated. The aim of this study is to prove that the SA-based smart samplers work, including that SA can be immobilized on oxidized filter paper and subsequently bind bmAbs. A combination of visual evaluation using a biotinylated fluorescence tag and LC–MS/MS-based experiments was used to evaluate immobilization of SA and the bmAb binding. Furthermore, quantitative performance of the optimized SA-based smart sampler was evaluated with hCG spiked to human serum samples to gather information of the potential of the sampler for quantitative analysis of biomarkers in biological samples.

## Experimental

### Chemicals and reagents

Acetonitrile (ACN, 99.9%), hydrochloric acid (37%), methanol (MeOH, LC–MS grade), potassium dihydrogen phosphate (≥ 99.5%), sodium azide (≥ 99%), sodium borate decahydrate (≥ 99.5%), sodium chloride (for analysis), and sodium dihydrogen phosphate monohydrate (≥ 99%) were purchased from Merck (Darmstadt, Germany). Ammonium hydrogen carbonate (ABC, ≥ 99.5%), biotin-5-fluorescein (B5F, ≥ 90%), bovine serum albumin (BSA, ≥ 96%), dl-dithiothreitol (DTT, ≥ 99.5%), iodoacetic acid (IAA, ≥ 98%), potassium chloride (≥ 99.9%), potassium periodate (≥ 99.8%), sodium dihydrogen phosphate dihydrate (≥ 98.5%), streptavidin from *Streptomyces avidinii* (> 13 units, 65–100%), Trizma® base (≥ 99.5%), trypsin from bovine pancreas TPCK treated (≥ 10,000 BAEE units/mg protein), and Tween 20® (≥ 99.5%) were purchased from Sigma-Aldrich (St. Louis, MO, USA). Biotinylated monoclonal anti-hCG antibody (bmAb; mouse) was purchased as Elecsys HCG STAT kit from Roche (Basel, Switzerland). The Elecsys HCG STAT kit contained biotinylated mAb against intact hCG with a concentration of 2.3 mg L^−1^ in phosphate buffer which was not further modified except for dilution with 50 mM ABC where applicable (“Optimization of bmAb amount” section). Formic acid (FA, for MS, ≥ 99%) was purchased from VWR International (Radnor, PA, USA). Water/MQW used in this project was filtered through a Merck Millipore Milli-Q integral water dispenser (resistivity of 18.2 MΩ cm). The monoclonal antibody E27 was donated by the Department of Medical Biochemistry, Oslo University Hospital (Oslo, Norway). E27 is a non-biotinylated monoclonal antibody against intact hCG and was previously described by Lund et al. [20]. Stable isotope-labeled AQUA™ peptide for hCG β peptide sequence (VLQGCLPALPQVVCNY[R_^13^C_6__^15^N_4_]) was used as internal standard (IS) and purchased from Sigma-Aldrich. The AQUA™ peptide was reduced and alkylated in-house according to the protocol from Lund et al. [20]. Ovitrelle® (Merck, London, UK) was used as hCG source and purchased from the local pharmacy.

### Fabrication and oxidation of paper discs

GE Healthcare Life Sciences (Buckinghamshire, UK) Whatman® grade 1 filter paper (pore size approx. 10 μm) was punched using a Philip Harris (Birmingham, UK) Uni-Core 6.0 mm puncher. For oxidation, the paper discs were incubated in 0.03 M aqueous potassium periodate solution at 65 °C for 2 h. Afterwards, the paper discs were washed with MQW, singled out, and dried overnight in a desiccator.

### Immobilization of streptavidin on oxidized paper discs

All immobilizations were carried out as follows (except for visualization experiments, conditions below): 5 µL 2% (w/v) SA in 1 mM HCl was applied to the paper discs which resulted in wetting of the whole disc. The immobilization was carried out overnight at room temperature in a sealed 96-well plate. The paper discs were then washed in triplicates in a tube filled with 1.5 mL 10 mM HCl followed by 0.1 M Tris HCl (pH 8), each time for 5 min on a Life Technologies™ AS (Norway) HulaMixer®. Afterwards, the discs were blotted on tissue and air-dried thoroughly until further procedure. In experiments where BSA was immobilized instead of SA, 5 µL 2% (w/v) BSA in 1 mM HCl was used.

### Visualization using biotin-5-fluorescein

For the visualization of bound SA, paper discs were immobilized with 0.5 µL 2% (w/v) SA overnight, washed, and dried. This volume was chosen to achieve a distinctive spot with a diameter less than that of the disc (6 mm). Controls were oxidized paper discs and discs which were immobilized with 0.5 µL 2% (w/v) BSA overnight; both sets were washed and dried subsequently. All discs were incubated with 3 µL 0.1% (w/v) B5F at room temperature for 30 min in a sealed 96-well plate. Triplicates of discs were washed three times in tubes filled with 1.5 mL 0.1 M Tris HCl (pH 8) for 5 min on a HulaMixer® and blotted on tissue between each washing. The discs were then left to air-dry at room temperature and visually evaluated using a UV lamp at 254 nm. In addition to visual evaluation, we used the software ImageJ (according to Pizzi et al. [17]) to calculate the fluorescence intensity of photographed spots under the UV lamp (Supplementary Material).

### Binding of antibody

Dried SA discs were placed individually in 1.5-mL tubes and variable volumes of 2.3 mg L^−1^ antibody solution were added to the various tubes (depending on the experiment between 15 and 750 µL). The incubation time was 30 min on an Eppendorf (Hamburg, Germany) ThermoMixer® set to 1300 rpm and operated at room temperature. After incubation, the discs were blotted on a tissue and triplicates were washed in 1.5 mL PBS with 0.05% (w/v) BSA for 5 min on a HulaMixer® followed by blotting and air-drying.

### Capture of hCG

To capture hCG, each disc was placed in a well of a 96-well plate. The discs were spotted with 5 µL hCG solution and the plate was sealed and incubated for 1 h at room temperature. The seal was removed, and the discs were left to dry for 45 min in the well plate while turning them in 15-min intervals. hCG solutions were prepared at varying concentrations depending on the experiment. After incubation, the discs were washed in three different steps each time in triplicates in 1.5 mL solution for 5 min on the HulaMixer®. In the first washing step, PBS with 0.05% (v/v) Tween 20 is used, the second 100 mM PBS, and the last 50 mM ABC. Blotting on tissue to remove access liquid was carried out between each washing step. Finally, the discs were left to air-dry until further procedure.

### Preparation of hCG samples

For the capturing step, hCG samples were prepared according to the experimental setup. Experiments without background matrix were done with hCG diluted to the desired final concentration using 50 mM ABC. When BSA was used as background matrix, hCG was diluted in 50 mM ABC containing 5% (w/v) BSA. Serum samples were prepared with 99% (v/v) serum and 1% (v/v) hCG which was diluted in 50 mM ABC, while control serum samples consisted of 99% (v/v) serum and 1% (v/v) 50 mM ABC.

### Human serum

Human serum from healthy blood donors (stored at − 30 °C) was obtained from Oslo University Hospital Ullevål (Oslo, Norway).

### Reduction, alkylation, and tryptic digestion

The prepared discs, depending on the respective experiment with or without mAb and captured hCG, were placed in a 1.5-mL tube and covered with 250 µL 50 mM ABC. The reduction was conducted by adding 3 µL 50 mM DTT and incubating at 60 °C for 15 min at 800 rpm. The samples were left to cool to room temperature and 3 µL 250 mM IAA was added for alkylation. The samples were agitated in the dark at room temperature for 15 min. For tryptic digestion, 10 µL of 1 mg mL^−1^ (in 50 mM ABC) trypsin was added to each sample and incubated at 37 °C for 16 h at 800 rpm. In case internal standard was used, it was added after digestion prior to LC–MS/MS analysis.

### LC–MS analysis and data interpretation

Chromatographic separation was carried out with a Dionex (Sunnyvale, CA, USA) Ultimate 3000 RSCL Nano system equipped with an Acclaim PepMap™ 100 C18 (ID: 75.0 µm, *L*: 150 mm, *d*_p_: 3.0 µm) separation column. A sample volume of 5 µL was loaded onto a Thermo Fisher Scientific (Waltham, MA, USA) Acclaim PepMap™ 100 C18 trap column (ID: 30.0 µm, *L*: 100 mm, *d*_p_: 5.0 µm) over 3 min at 10 µL min^−1^ with 20 mM FA/ACN (2:98, v/v). Mobile phase A consisted of 20 mM FA/ACN (95:5, v/v) and mobile phase B of 20 mM FA/ACN (5:95, v/v). The separation flow was set to 300 nL min^−1^ and column temperature to 35 °C. The gradient started after a delay of 5 min at 95% mobile phase A and ramped up over 25 min to 55% mobile phase B. This was followed by 5 min 95% mobile phase B and then back to starting conditions. The LC system was coupled to a Thermo Fisher TSQ Quantiva triple quadrupole operated in positive SRM mode with Nano ESI at 2.0 kV applied voltage. Acquisition was carried out with the following parameters: ion transfer tube temperature: 350 °C, collision energy: 30 V, CID gas pressure: 1.5 mTorr, 1s scan cycle, and Q1 and Q3 resolutions at 0.7 FWHM. Two peptides were monitored: the βT5 peptide (sequence: VLQGCLPALPQVVcNYR) and the internal standard peptide (VLQGCLPALPQVVcNY [R_^13^C_6__^15^N_4_]). For the βT5 peptide, the following two transitions were monitored: *m*/*z* 964.2 → 1036.3 and *m*/*z* 964.2 → 1317.8. Similarly, for the internal standard peptide, the transitions *m*/*z* 969.2 → 1046.3 and *m*/*z* 969.2 → 1327.8 were monitored. Data acquisition and processing were carried out using Thermo Fisher Scientific Xcalibur™ software.

## Results and discussion

### Visual characterization of streptavidin immobilization

Periodate treatment of cellulose filter paper oxidizes the cellulose’s hydroxyls to aldehyde groups which enables a reaction with the amine groups of the protein to be immobilized [21]. Studies have already shown the possibility of immobilizing molecules to oxidized paper, such as trypsin still possessing its proteolytic abilities [17]. Based on such experiments, the question was raised whether it is possible to immobilize streptavidin on oxidized paper as a potential universal anchor to bind biotinylated molecules. In preliminary experiments, the covalent binding of SA to oxidized filter paper was investigated through coupling a biotinylated fluorescent tag, biotin-5-fluorescein (B5F), to it. The presence of B5F was visually evaluated under UV light (at 254 nm).

First, the best conditions for visualization of SA immobilization were established: The effect of SA concentrations in the range of 0–2% (w/v) on the fluorescence was investigated (Supplementary material, Fig. S1). From 0.5% (w/v) SA and higher, there was no increase in the fluorescence intensity. Furthermore, different concentrations of B5F in a range from 0.01 to 0.5 mg mL^−1^ were assessed to determine the amount of biotin bound to SA paper discs. Already low concentrations of 0.05 mg mL^−1^ showed a saturation (Supplementary material, Fig. S2). Based on these results, the conditions to be used to visualize SA immobilization were decided to be 0.5 µL 2% (w/v) SA and a B5F concentration of 0.1 mg mL^−1^. This is not only to ensure a saturation of SA on the oxidized paper but also saturation of SA with B5F.

**Fig. 1:**
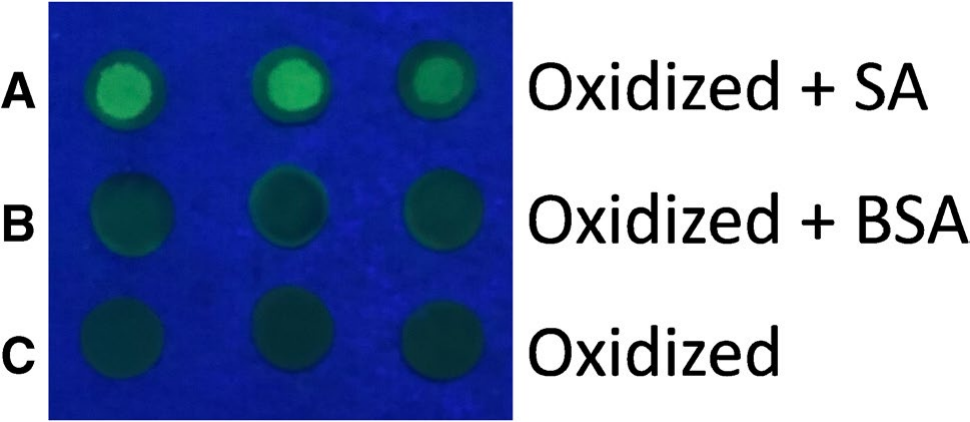
Photograph of paper discs under UV light (254 nm, *n* = 3): **A** oxidized paper treated with 2% (w/v) 0.5 µL streptavidin (SA) and incubated with biotin-5-fluorescein (B5F), **B** oxidized paper treated with 0.5 µL 2% (w/v) bovine serum albumin (BSA) and subsequent incubation with B5F, and **C** oxidized paper discs directly incubated with B5F

After establishing optimal conditions for visualization, the immobilization of SA was evaluated. For that, three different types of paper samplers have been prepared: paper which was oxidized (blanks), paper which was oxidized and immobilized with streptavidin, and paper that was oxidized and treated with BSA. For the latter two, a volume of only 0.5 µL of protein (SA or BSA) was applied in order to leave a distinct spot on the discs. Figure 1 shows SA paper treated with B5F (Fig. 1A) opposed to the controls: oxidized paper discs without SA (Fig. 1C) and oxidized paper discs immobilized with BSA instead of SA (Fig. 1B) all incubated with B5F, accordingly. Only paper discs that have been oxidized and subsequently immobilized with SA (Fig. 1A) emit strong fluorescence after incubation with B5F. On paper discs, which were oxidized and immobilized with BSA (Fig. 1B) or blanks (Fig. 1C), only residual fluorescence was detected. For paper discs which were immobilized with SA, a distinct circle emitting fluorescence can be seen. This shows the successful binding of B5F (Fig. 1A) to the discs, indicating successful immobilization of SA to the oxidized paper. For BSA-treated and oxidized paper discs (Fig. 1B, C, respectively), the whole circumference shows low emission of fluorescence. This indicates that B5F molecules did not bind to BSA (Fig. 1B) nor to oxidized paper, (Fig. 1C), but were bound nonspecifically to BSA or adsorbed to the oxidized paper, respectively. While oxidized paper without SA or BSA shows a homogenous distribution of residual fluorescence (Fig. 1C), paper discs containing BSA show a slight “coffee ring effect” (Fig. 1B). This phenomenon describes the evaporation of sessile droplets containing non-volatile solutes drawing water from the inside of the droplet to the outside, leaving a circle-shaped stain [34]. Based on these experiments, it was concluded that immobilization of SA to the oxidized paper was successful under the given conditions.

### Binding of bmAb to SA-immobilized paper

**Fig. 2:**
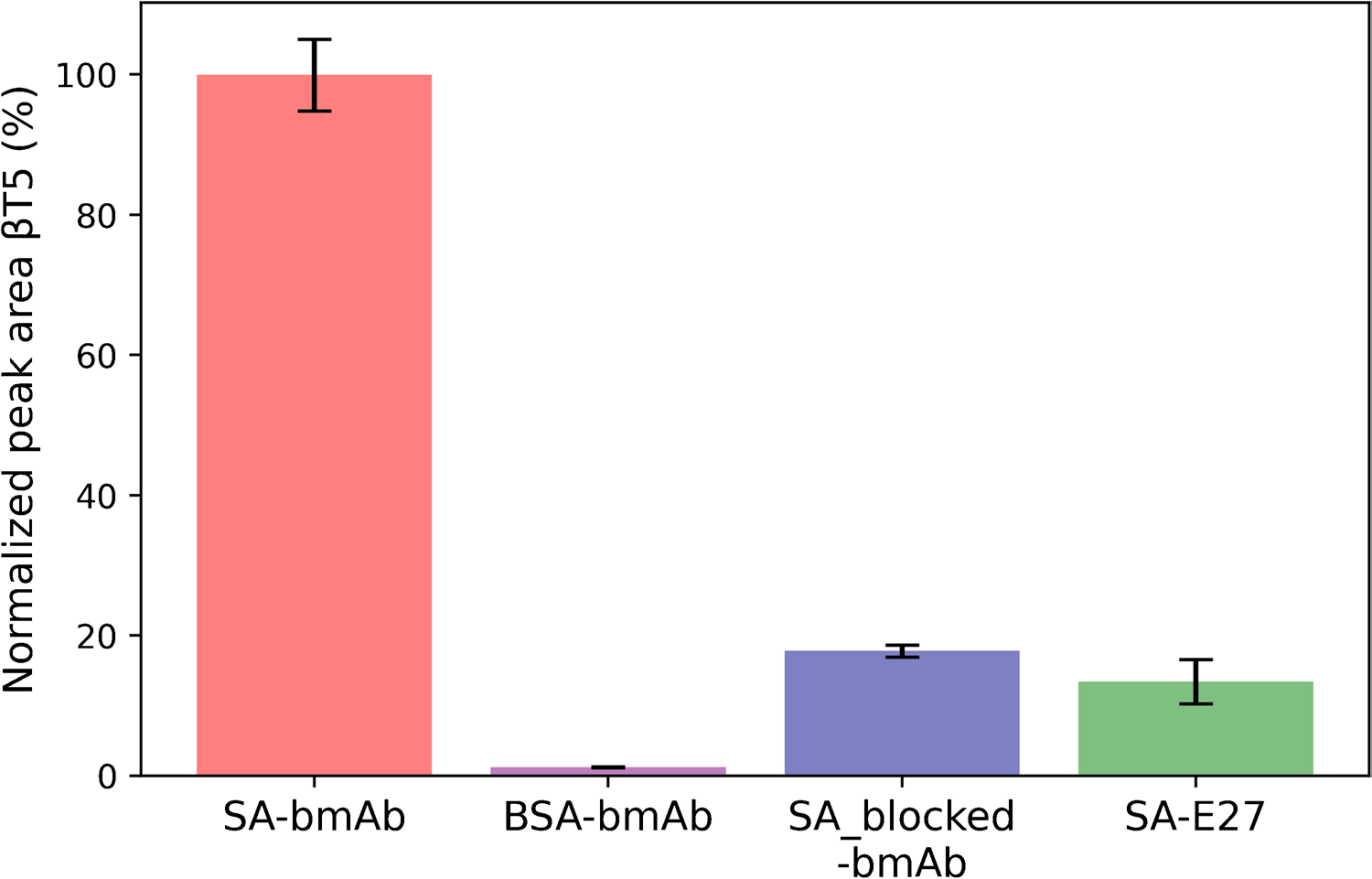
Normalized peak areas of hCG’s βT5 peptide in four different conditions (*n* = 3). SA-bmAb: Samplers were incubated with SA, added bmAb, and used for hCG capture. BSA-bmAb: Samplers were incubated with 2% BSA, added bmAb, and used for hCG capture. SA_blocked-bmAb: Samplers were incubated with SA, added (blocked with) B5F, followed by bmAb incubation, and used for hCG capture. SA-E27: Samplers were incubated with SA and added mAb E27 (non-biotinylated), and used for capture of hCG. For all conditions 5 µL 100 ng mL^−1^ hCG in 50 mM ABC, 5% BSA was used as sample. 250 µL antibody (bmAb and mAb) in a concentration of 2.3 mg mL.^−1^ was used. The peak areas were normalized to the response of hCG captured on the SA-bmAb samplers (= 100%)

After successfully immobilizing SA to oxidized paper, its ability to bind biotinylated antibody was investigated. For this, bmAb against the model protein hCG was used to target the intact form of hCG. For proof of principle, paper was immobilized with 2% (w/v) SA and subsequently treated with 250 µL of 2.3 mg L^−1^ bmAb. The modified paper was then incubated with 5 µL hCG (100 ng mL^−1^, in 50 mM ABC; Fig. 2, SA-bmAb). After capturing hCG, the discs were washed and then subjected to reduction, alkylation, and tryptic digestion. The mass spectrometric detection of the βT5 peptide of hCG in the digestion solution concluded that hCG was present. To confirm that the proposed mechanism works, three different control conditions were investigated. For all conditions, 250 µL of 2.3 mg L^−1^ mAb (biotinylated or non-biotinylated depending on the condition) was applied and incubation with the target analyte performed using 5 µL of 100 ng mL^−1^ hCG in 50 mM ABC. Firstly, it was tested if hCG could be captured with oxidized paper discs immobilized with BSA (5 µL of 2% (w/v) in 1 mM HCl) instead of SA (Fig. 2, BSA-bmAb). For the second condition (Fig. 2, SA_blocked-bmAb), SA paper was treated with an excess amount of B5F (regarding SA) to block SA binding sites prior to adding the bmAb. The last condition (Fig. 2, SA-E27) was characterized by using a non-biotinylated hCG antibody (E27) in the antibody binding step (250 µL, 2.3 mg L^−1^). These conditions were compared to the proof-of-principle procedure (Fig. 2, SA-bmAb). As can be seen in Fig. 2, the proof-of-principle procedure (SA-bmAb) showed the highest signal for the βT5 peptide compared to any of the control conditions. Paper discs which were immobilized with BSA instead of SA prior to the binding of bmAb and capture of hCG showed the lowest signal for the βT5 peptide. Compared to SA-bmAb, the BSA discs showed a recovery of 1.2 ± 0.1% for hCG capture (Fig. 2, BSA-bmAb). This indicates that SA as an anchor for the bmAb is essential for antibody binding and that the bmAb only shows a low degree of nonspecific binding. The very low signal for hCG (1.2 ± 0.1% recovery) on BSA-immobilized paper indicates that BSA bound to the oxidized paper and eliminated most of the nonspecific binding sites: BSA immobilization prevents nonspecific binding of the bmAb (and subsequent capture of hCG) and hCG to the paper. Blocked SA (using B5F), as well as the usage of non-biotinylated mAb (E27), showed comparable outcomes of 13.4 ± 3.2% and 17.7 ± 0.9%, respectively. The modest recovery for blocking of SA using B5F (13.4 ± 3.2%) can be due to insufficient conditions for the blocking or nonspecific binding of either bmAb or hCG to the paper. In case that the conditions for blocking SA with B5F were insufficient, unblocked SA would still be able to bind bmAb, which in turn binds hCG and therefore produces a signal. If the blocking was sufficient and the moderate recovery is due to nonspecific binding, then this can be caused by either bmAb (binding hCG) or hCG itself. Using a non-biotinylated mAb also shows a modest signal which could result from nonspecific binding of either the mAb or hCG to the paper. Overall, the results depicted in Fig. 2 support the hypothesis that immobilized SA binds bmAb and that this is essential for the successful capture of hCG. It also excludes that the produced signal for the βT5 peptide in the proposed procedure is primarily caused by nonspecific binding. It is therefore concluded that bmAbs can be successfully bound to SA treated paper and that the target analyte is majorly captured by the bmAb.

### Optimization of bmAb amount

**Fig. 3:**
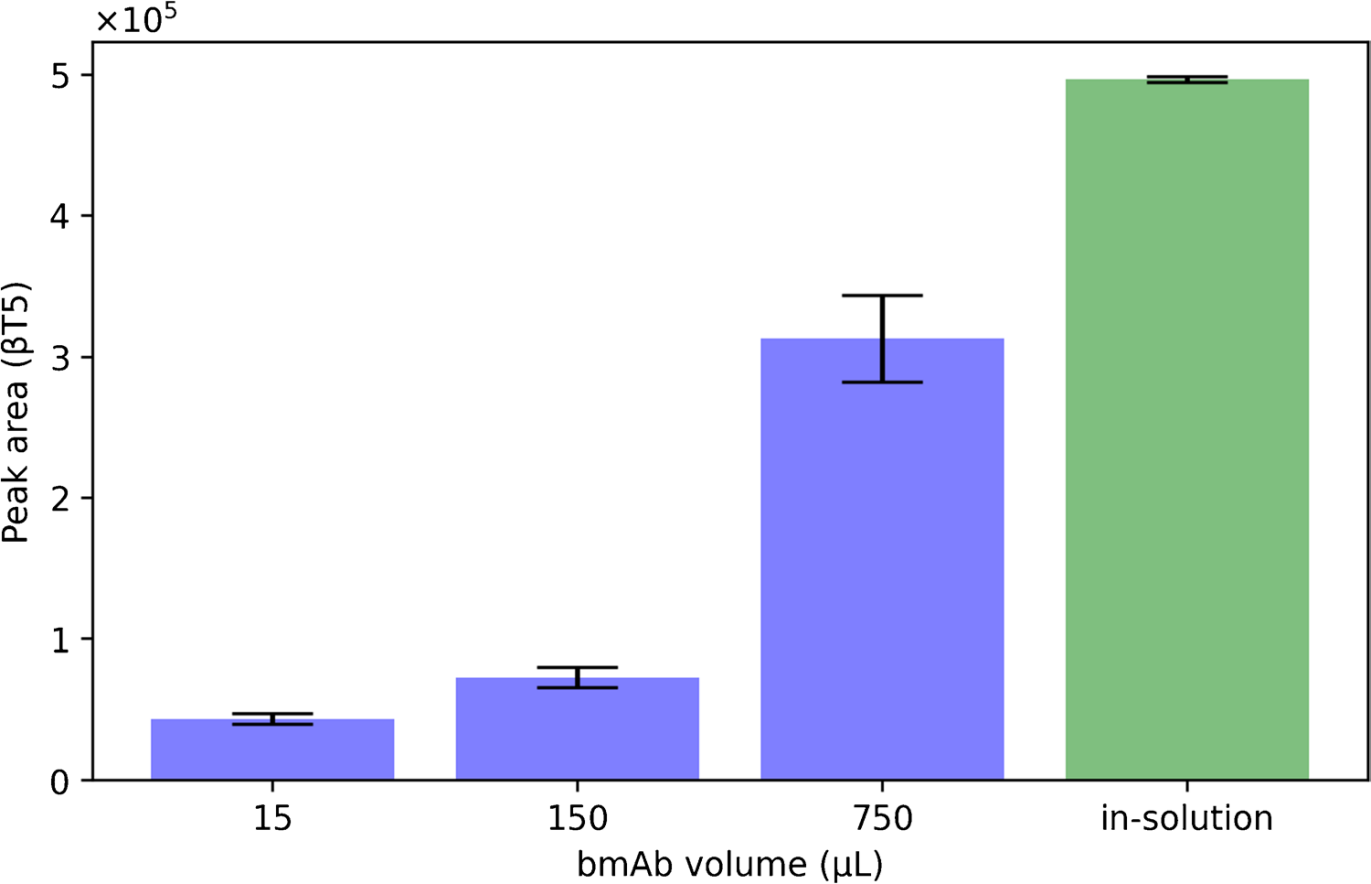
Different bmAb amounts used during immobilization on SA treated paper discs, and subsequent capture of 5 µL 1 µg mL.^−1^ hCG in ABC, containing 5% (w/v) BSA (*n* = 3)

In order to evaluate whether the amount of bmAb was limiting the hCG binding and to ensure high recoveries of the target analyte, paper discs were treated with varying volumes of bmAb (15, 150, and 750 µL) while the hCG concentration remained constant (1 µg mL^−1^ in 50 mM ABC with 5% (w/v) BSA). Theoretically, all three volumes of bmAb contained sufficient amounts of antibody to capture the entire amount of hCG added (for calculation, see supplementary material, Table S1). However, an increase in peak area with increasing amount of bmAb was observed (Fig. 3). This increase cannot be explained by insufficient amounts of antibody. One potential explanation could be a low binding capacity of the bmAb. Lund et al. reported a 32–43% recovery for hCG in serum samples and 57–65% for urine samples when hCG were extracted using mAbs coated to magnetic beads [20]. In this study’s experimental setup, 5% (w/v) BSA was used as background matrix and extraction was not performed on beads; however, the modest recovery of hCG could be related. As comparison, an in-solution digest of 5 µL 1 µg mL^−1^ hCG in 50 mM ABC with 5% (w/v) BSA (no paper disc; Fig. 3, “in-solution”) was conducted representing the maximal possible peptide turnout for the applied amount of hCG. Assuming that the in-solution digest has a theoretical efficacy of 100% for the production of βT5 peptides, then the highest amount of bmAb used (750 µL, 1725.0 ng) had a recovery of 63 ± 6% of the applied hCG. The smallest amount (15 µL, 34.5 ng) had a recovery of 8.7 ± 1% and the second highest amount (150 µL, 345.0 ng) 14.6 ± 1%. Even though the recovery is not directly proportional to the used amount of bmAb, a trend can be seen. The higher the applied amount of bmAb, the higher recovery for the βT5 peptide. This implies that low binding capacity of the bmAb might not be the only effect taking place. Additional explanations could be a low binding of bmAb on the SA paper or loss of bmAb during preparation, for example, nonspecific binding to tubes and pipette tips. If the amount lost is constant, these effects have a bigger impact at low concentrations. In conclusion, the theoretical amount of bmAb used is sufficient to capture the applied amount of target analyte; however, empirical data shows otherwise. To find a compromise of optimal capture efficiency and cost-effectiveness, further experiments were conducted using 150 or 250 µL (2.3 mg L^−1^) bmAb.

### Evaluation of hCG binding to bmAb paper

**Fig. 4:**
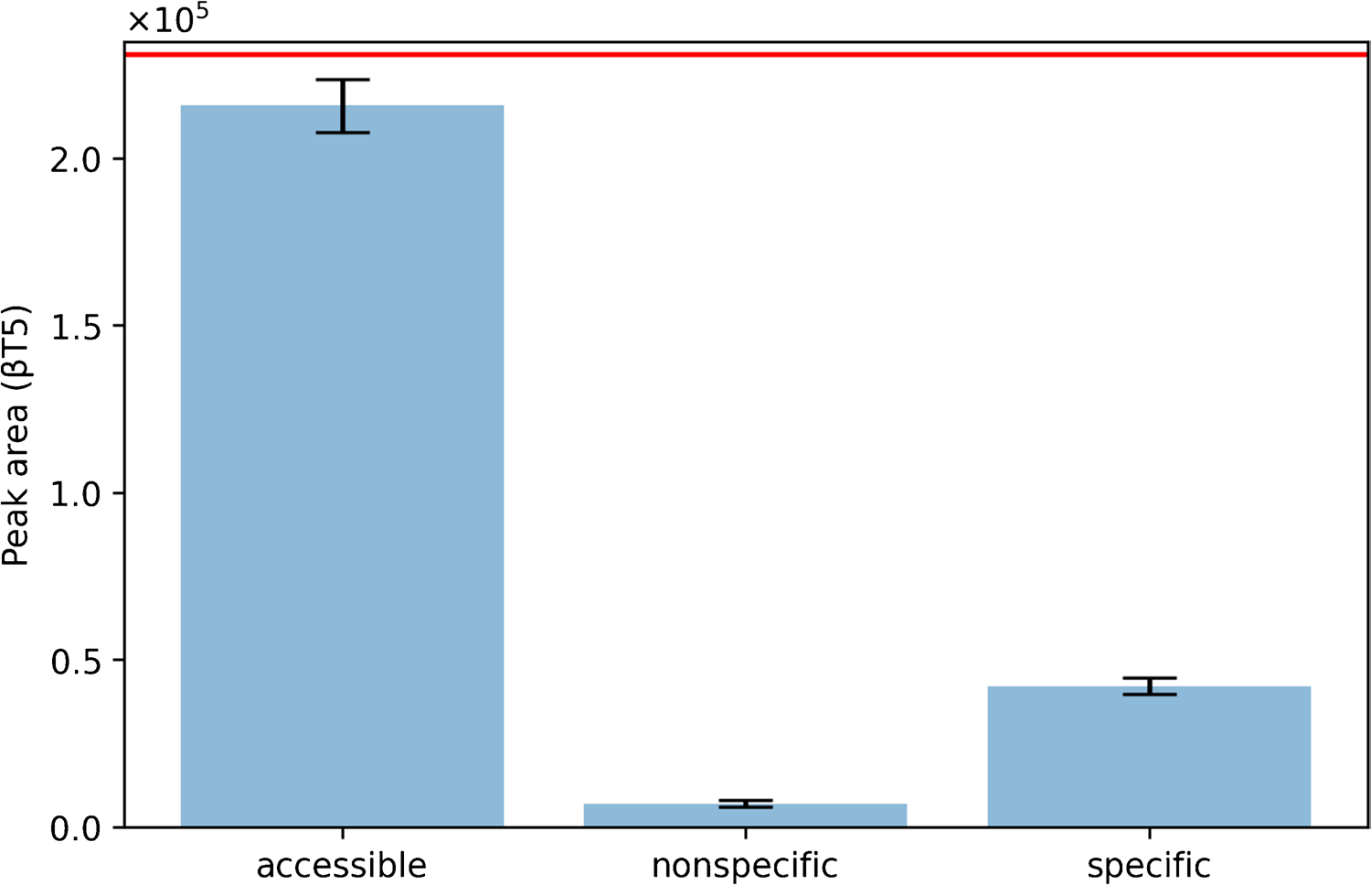
Evaluation of nonspecific and specific binding in comparison to the accessible amount (*n* = 3). Antibody immobilization was carried out with 250 µL (2.3 mg mL^−1^) bmAb and 5 µL 100 ng mL^−1^ hCG in ABC; 5% BSA was added. The red line indicates the average peak area for an in-solution digest of 5 µL 100 ng mL.^−1^ hCG in ABC, 5% BSA

Above, the performance of the procedure was amongst others evaluated by comparing it to an in-solution digest. This comparison implies that the entire volume of hCG solution is absorbed by the paper during the capturing step, that all hCG molecules are bound by the bmAb, and also that it is available for digestion. Due to low binding capacity of the bmAb for hCG and/or loss of bmAb or analyte during preparation (see the “Optimization of bmAb amount” section), this assumption is flawed. Therefore, we investigated nonspecific and specific binding of hCG to bmAb-treated paper and compared it to the accessible hCG amount available from the paper discs. Not all hCG may be captured by the antibodies and part of it might be washed off by the subsequent washing steps; therefore, the experiment was designed to show the differences in hCG capture on bmAb-treated versus bmAb-untreated paper (specific vs. nonspecific, respectively) and the impact of the washing procedure. To determine the specific binding, samples were prepared using SA paper discs treated with bmAb and incubated with hCG (250 µL bmAb, 5 µL of 100 ng mL^−1^ hCG in 50 mM ABC, containing 5% (w/v) BSA, respectively; Fig. 4, specific). The nonspecific binding was measured by applying hCG (5 µL of 100 ng mL^−1^ in 50 mM ABC, 5% (w/v) BSA) on SA paper discs not treated with bmAb, but otherwise following the standard procedure (Fig. 4, nonspecific). The accessible amount was determined from a sample (same amount of hCG) applied to SA-immobilized paper without bmAb which was not washed after hCG capture (Fig. 4, accessible). Figure 4 shows peak areas for the βT5 peptide of hCG for the three different conditions. It can be seen that the peak area for the non-washed sample (accessible amount) is approx. 3 times higher than the peak area for the hCG added to the bmAb-treated paper (specific binding portion). This means that approx. 67% of the accessible hCG is possibly washed off the paper during the washing step or lost during sample application (assuming equal digestion efficiency). In turn, a recovery of captured hCG of 33% can be estimated which is congruent with the findings of Lund et al. [20], and previous results in the “Optimization of bmAb amount” section. The results shown in Fig. 4 also indicate that using immunocapture, a 14-fold increase in target binding compared to binding without bmAb is achieved. Comparing the accessible amount to the nonspecific binding amount, nonspecific binding makes 2% of the accessible amount (Fig. 4). This implies that the washing step is highly efficient in removing adsorbed protein, and it confirms that the signal observed for the SA-bmAb samplers is mainly due to specific interaction between the bmAb and hCG. A final comparison can be made between the accessible amount and an in-solution digest. This comparison gives information about not only adsorption of hCG or its peptides to the paper making them possibly unavailable for further analysis but also loss of the analyte during its application on the paper. It can be assumed that part of the hCG remains in the well after spotting it on the paper since the volume (5 µL) is not fully absorbed at once by the SA paper discs. Therefore, comparing the accessible amount (hCG spotted on SA-bmAb paper but not washed) to an in-solution digest (hCG spiked in digestion solution) can give an indication on how great the impact is. The signal for the accessible amount makes approximately 85% of the signal of the in-solution digest for the same amount of hCG (Fig. 4, red line). If the remaining 15% are lost during preparation or not available for digestion, or both, cannot be concluded.

### Application in complex samples

**Fig. 5:**
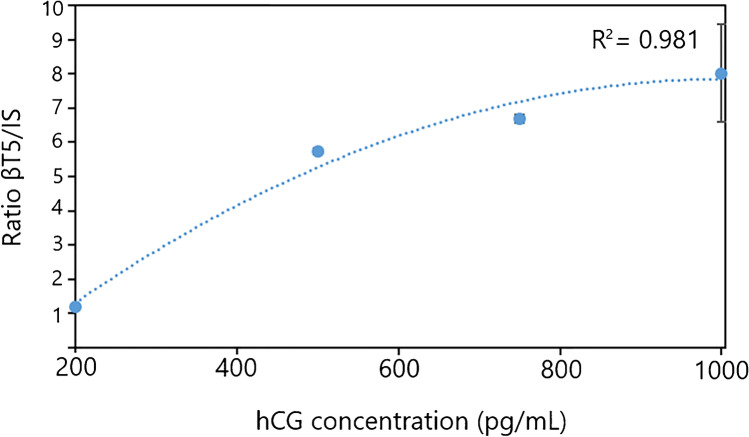
Four-point concentration curve for different hCG concentrations in 99% human serum (*n* = 3) spiked with internal standard. SA paper treated with bmAb was incubated with hCG in the concentrations 200 pg mL^−1^, 500 pg mL^−1^, 750 pg mL^−1^, and 1000 pg mL^−1^. After digestion, the samples were spiked with 5 pmol mL^−1^ IS. Polynomial regression was obtained to be *R*.^2^ = 0.98

**Fig. 6:**
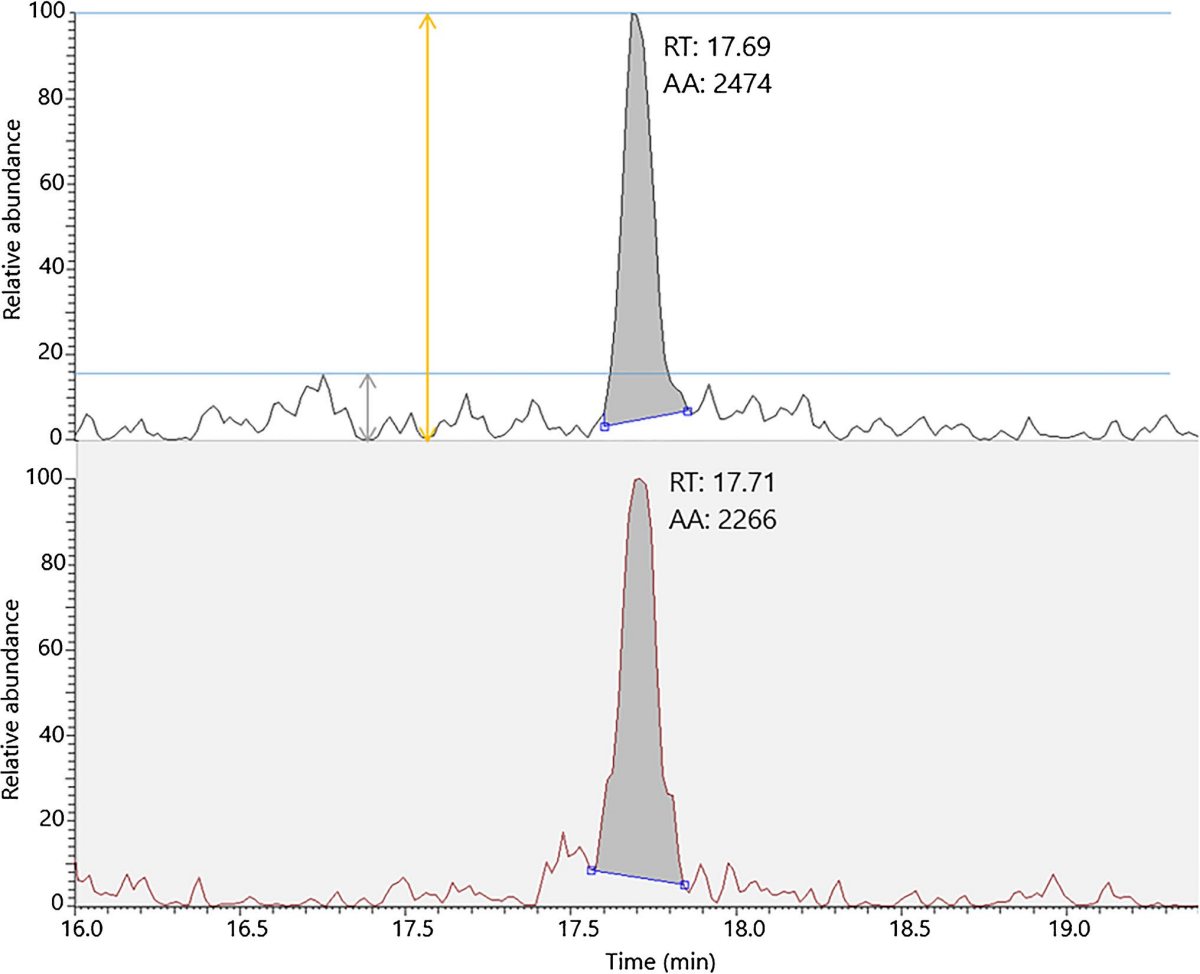
Extracted ion chromatograms (EIC) for the βT5 peptide from SA paper treated with bmAb, subsequent capture of 200 pg mL.^−1^ hCG spiked in human serum and addition of IS. (i) EIC of the βT5 peptide from Ovitrelle®. (ii) EIC of the βT5 peptide of the IS

Since the described bmAb-SA papers are intended for sampling and subsequent analysis of complex human samples similar to DBS, an evaluation of the performance using target analyte spiked in human serum was carried out. SA paper discs were treated with 150 µL, 2.3 mg L^−1^ bmAb with subsequent capture of 5 µL hCG in different concentrations spiked in human serum. The digested samples were spiked with 5 pmol mL^−1^ of the internal standard (IS). Figure 5 shows the ratio of the βT5 peak area of the samples divided by the βT5 peak area of IS with increasing concentration (200–1000 pg mL^−1^) of hCG. The correlation value (*R*^2^) for the polynomial regression equals 0.98. The relative standard deviation (RSD) of each concentration was 7.0%, 7.0%, 9.1%, and 8.7% for 200 pg mL^−1^, 500 pg mL^−1^, 750 pg mL^−1^, and 1000 pg mL^−1^, respectively. The calibration curve in Fig. 5 indicates that a saturation might be reached at the highest hCG concentration (1000 pg mL^−1^) for the used amount of bmAb (150 µL, 2.3 mg L^−1^). This could be a possible explanation for the modest correlation value. However, it is shown that different hCG concentrations can be extracted from human serum when using the tailor-made affinity smart samplers. The average signal to noise ratio (*S*/*N*) for the 200 pg mL^−1^ samples (*n* = 3) is 6.1. Assuming a *S*/*N* = 3 as definition of the limit of detection (LOD), then an estimate of 65 pg mL^−1^ can be determined for SA-bmAb paper discs (Fig. 6). To further improve the LOD or limit of quantification for extracted hCG, the amount of bmAb could be optimized to increase the capacity for target analyte capture. However, it can be concluded that the smart samplers can be used for complex human samples.

## Conclusion

This study demonstrated the functionality of a novel immunocapture smart sampler device on paper. Fluorescent visualization of SA binding to KIO_4_-functionalized paper showed that SA was successfully immobilized. The investigations of interactions between SA and the bmAb confirmed that the bmAb was bound to SA paper, and able to capture the target analyte. Furthermore, it was shown that the majority of target analyte was bound specifically. A recovery of 33% of specifically bound target analyte was obtained for slightly optimized conditions. The application to human serum samples indicates that the immunocapture smart sampler can be used for patient samples as well, with an estimated LOD of 65 pg mL^−1^ for capturing hCG on SA-bmAb paper discs. Further optimization needs to be done to show the full versatility of the device, however; this study successfully demonstrated that the principle works. Furthermore, the concept of utilizing SA as a universal anchor for biotinylated antibodies is not limited to hCG but might easily be exchanged with any other bmAb to capture different target analytes. Even though bottom-up LC–MS is a sophisticated analytical technique which calls for special equipment and infrastructure, combining simple sample collection and sample pretreatment on an easily available material like paper shows potential for an application in resource-poor regions.

## Supplementary Information

Below is the link to the electronic supplementary material.

Supplementary file1 (DOCX 30.5 KB)

## Declarations

### Source of biological material

Serum from healthy blood donors was obtained from Oslo University Hospital, Oslo, Norway.
