# Supplementary material for: On the spot immunocapture in targeted biomarker analysis using paper-bound streptavidin as anchor for biotinylated antibodies
Source: Anal Bioanal Chem. 2022 Jun 10;414(19):5979–89. doi: 10.1007/s00216-022-04161-w (PMC9293813; doi:10.1007/s00216-022-04161-w)
Supplement: Supplementary file 1 — Supplementary file1 (DOCX 30.5 KB) [file 216_2022_4161_MOESM1_ESM.docx]

**Supplementary Material**

On the spot immunocapture in targeted biomarker analysis using paper-bound streptavidin as anchor for biotinylated antibodies

Christina Johannsen^1^, Anam ul haq^2^, Léon Reubsaet^2^, Trine Grønhaug Halvorsen^2^

^1^Section of Biochemistry and Molecular Biology, Department of Biosciences, University of Oslo, Oslo, Norway

^2^Section of Pharmaceutical Chemistry, Department of Pharmacy, University of Oslo, Oslo, Norway

## S1 Optimization of SA concentration

0.5 µL SA in the concentrations 0 %, 0.05 %, 0.1 %, 0.5 %, 1 % and 2 % were immobilized on oxidized filter paper to evaluate the optimal binding conditions for SA. The discs were incubated overnight in a sealed 96-well plate, followed by washing with 15 µL of 10 mM HCl and left to air-dry. The dried SA discs were subsequently incubated with B5F, which binds to SA and therefore indicates its binding to the paper disc. The discs were evaluated visually with a UV-lamp as well as with the online tool ImageJ. ImageJ has been operated according to the protocol of Pizzi *et al.* [21]. Fig. S1 shows the measured intensity plotted against the SA concentration.

**Fig. S1** Graphical presentation of concentration gradient of SA, where average values of intensity (middle green lighted areas of discs) measured by ImageJ are on the Y-axis and concentration of SA (in % (w/v)) are on the X-axis.

S2 Optimization of B5F concentrations

SA treated paper discs were incubated with B5F in the concentrations 0.01 mg·mL^-1^, 0.05 mg·mL^-1^, 0.1 mg·mL^-1^, 0.5 mg·mL^-1^ and 1 mg·mL^-1^. The discs were incubated for 30 min in a sealed 96-well plate and washed 10 times with 15 µL 0.1 M Tris HCl buffer. The discs were air-dried and evaluated visually under a UV-lamp emitting light of 254 nm and the intensities of the spots were determined using ImageJ. Fig. S2 shows the measured intensity plotted against the B5F concentration.

**Fig. S2** Graphical presentation of concentration gradient of B5F, where average values of intensity (middle green lighted areas of discs) measured by ImageJ are on the Y-axis and concentration of B5F in mg/ml is on the X-axis.

## S3 bmAb calculations

Theoretically, every molecule of SA has four binding sites for biotinylated molecules. However, due to steric hindrance and other effects for binding of large biotinylated molecules, we assume in the following calculations that at least one bmAb can bind to one molecule of SA and at least bind one hCG molecule. In the initial experiments (Fig. 2), the concentration used for the SA immobilization (2 % (w/v)) results in 1666.7 pmol SA in case of 100 % recovery (Table S1) while 250 µL of 2.3 mg·L^-1^ used for the immobilization of bmAb results in 3.8 pmol (Table S1). For the capturing step in the experiment described above 5 µL of 100 ng·mL^-1^ hCG solution was used (0.5 ng per paper disc and therefore a molarity of 0.01 pmol - Table S1), respectively. Meaning, the amount of SA used is in large excess to the amount of bmAb (approx. 438 times) assuming one molecule of SA binds only one (and not four) bmAbs. Additionally, the amount of hCG used in the experiment is less than the theoretical binding capacity of the bmAb (3.8 pmol bmAb vs. 0.013 pmol hCG) assuming 100 % binding efficacy of bmAb to SA-paper, meaning that the capacity of the SA-bmAb paper should be sufficient for efficient hCG binding. Assessment of the effect of increasing amounts of bmAb was carried out using a considerably higher hCG concentration. It was chosen to be 5 µL of 1 µg·mL^-1^, resulting in 0.13 pmol per disc. Three different antibody amounts were evaluated 0.2 pmol, 2.3 pmol, and 11.5 pmol (Table S1). The lowest amount of antibody (0.2 pmol) resulted in a volume of 15 µL, which for practical reasons could not be lowered further. All three amounts of antibody are theoretically sufficient to capture the entire amount of hCG.

Table S1 Calculations for used amounts of SA, bmAb and hCG for different experimental set ups.

| **Substrate** | **Concentration** | **Volume (µL)** | **Amount per disc (ng)** | ***n* (pmol)** |
| --- | --- | --- | --- | --- |
| Streptavidin | 2 % (w/v) | 5 | 100000 | 1666.7 |
| Biotinylated anti-hCG antibody | 2.3 mg·L^-1^ | 250 | 575 | 3.8 |
| hCG | 100 ng·mL^-1^ | 5 | 0.5 | 0.01 |
|  | 1 µg·mL^-1^ | 5 | 5 | 0.13 |
| Calculations for **3.3 Optimization of bmAb amount** | | | | |
| bmAb | 2.3 mg·L^-1^ | 15 | 34.5 | 0.2 |
|  |  | 150 | 345 | 2.3 |
|  |  | 750 | 1725 | 11.5 |
